# Nasal carriage rate, associated factors, and antimicrobial susceptibility patterns of methicillin resistance *Staphylococcus aureus* among pre-clinical undergraduate students at the College of Health and Medical Sciences, Haramaya University, Ethiopia

**DOI:** 10.3389/fpubh.2024.1354461

**Published:** 2024-05-23

**Authors:** Fitsum Weldegebreal, Kedir Urgesa, Firayad Ayele, Kasahun Bogale, Taddese Shume, Mohammed Ahmed, Sileshi Debebe, Fikru Tebeje, Haftu Asmerom, Tewodros Tesfa, Shambel Mekonnen

**Affiliations:** ^1^School of Medical Laboratory Sciences, College of Health and Medical Sciences, Haramaya University, Harar, Ethiopia; ^2^Laboratory Bacteriology Research, Faculty of Medicine and Health Sciences, Ghent University, De Pintelaan, Belgium

**Keywords:** nasal carriage, *Staphylococcus aureus*, methicillin-resistant *Staphylococcus aureus*, students, Harar

## Abstract

**Background:**

*Staphylococcus aureus* nasal carriage has been linked to higher rates of infection and morbidity. People with Methicillin-resistant *Staphylococcus aureus* can be a potential source of infection for others. University students living together in crowded conditions increase their risk of acquiring infections. The prevalence of *S. aureus*, particularly Methicillin-resistant *Staphylococcus aureus* nasal carriage, in Ethiopian university students is sparse.

**Objective:**

This study aimed to determine the nasal carriage rate, associated factors, and antimicrobial susceptibility patterns of methicillin-resistant *Staphylococcus aureus* among pre-clinical students at the College of Health and Medical Sciences, Haramaya University, Ethiopia, from 1 July to 30 August 2022.

**Methods:**

An institutional-based cross-sectional study was conducted among 270 randomly selected pre-clinical Health and Medical Sciences students. Data on associated factors were collected using pre-tested, structured questionnaires. A nasal swab was taken from each participant and sent to the microbiology laboratory via Amies transport media in a cold chain. There, it was cultivated using conventional techniques. The isolated colonies were found to be *S. aureus*, and its antimicrobial susceptibility was performed using the Kirby–Bauer disk diffusion method on Muller–Hinton agar. Methicillin-resistant *Staphylococcus aureus* expressing using cefoxitin based on CLSI breakpoint. Data were entered into Epi-Data version 4.4.2.1 and exported to the Statistical Package for Social Sciences (SPSS) software version 25 for analysis. Pearson’s chi-square test was performed to predict the associations between variables. A *p*-value less than 0.05 was regarded as statistically significant.

**Result:**

Methicillin-resistant *Staphylococcus aureus* nasal carriage was 5.9% (95% CI: 3.09–8.7) of cases of *S. aureus* nasal colonization, which was found to be 12.96% (95% CI: 8.85–16.96). Methicillin-resistant *Staphylococcus aureus* nasal colonization was significantly associated with the history of cigarette smoking (*p* = 0.000), intake of khat (*p* = 0.042), nose-picking habit (*p* = 0.003), history of sharing personal goods (*p* = 0.021), and history of hospitalizations (*p* = 0.00). All of the Methicillin-resistant *Staphylococcus aureus* isolates were resistant to ampicillin and cefoxitin.

**Conclusion:**

Based on the findings, a considerable proportion of healthy students harbored Methicillin-resistant *Staphylococcus aureus* strains associated with behavioral factors. Furthermore, these isolates showed high resistance to cefoxitin and ampicillin. Hence, it is crucial to regularly test pre-clinical students to prevent endogenous infections and the spread of Methicillin-resistant *Staphylococcus aureus*.

## Introduction

### Background

*Staphylococcus aureus* is a prevalent bacterium that frequently causes nosocomial infections and public health issues (1, 2). Methicillin-resistant *Staphylococcus aureus* (MRSA) is now the main source of community-acquired infections among individuals who have not been exposed to healthcare environments (3). Nasal MRSA colonizes approximately 30% of the human population (4).

Individuals with a history of hospital admissions, history of nasal polyps, HIV/AIDS, malaria, tuberculosis, malnourishment, climate change, sharing of personal items without frequent cleaning, frequent skin-to-skin contact in skin infection, crowded housing, and MRSA carriage have all been linked to an increased risk of *S. aureus* and MRSA infections (3–9). The MRSA strains are typically spread through direct skin-to-skin contact and can occur in shared public areas such as dorms, gyms, and barracks (10, 11), causing community-acquired infections. According to reports, the chance of contracting MRSA might increase by 7.5 times when one is close to someone who has previously been colonized or infected (2, 7, 12, 13). MRSA infections have increased in people who have not been exposed to healthcare settings since the 1990s. Consequently, strains of community-associated methicillin-resistant *Staphylococcus aureus* (CA-MRSA) have been identified (14). Given the increasing evidence that MRSA acquired in the community is spreading among healthy individuals (15). Serious opportunistic infections in humans and systemic illnesses such as sepsis, infections related to healthcare, and infections of the skin, soft tissues, and bones are caused by *S. aureus* (16). It frequently results in major infections with significant rates of morbidity, mortality, and expenses related to medical care (11). Approximately 10–35% of the world’s population harbors MRSA in their anterior nares (17), and the prevalence of MRSA ranges from 5.0 to 73.0% (18). However, the burden of infection is high in developing countries (19). People with MRSA infections are 64% more likely to die than people with drug-sensitive infections (20). According to earlier research, the carriage rate of *S. aureus* in 2013 was 21% at Tanzanian universities (21), 22.1% at Jimma University in Ethiopia (22), and 27.1% at Arba Minch University in Ethiopia (7). These findings were significant for MRSA.

*S. aureus* strains have developed resistance to β-lactam antibiotics, including MRSA (23). The organism acquires resistance through the insertion of the mecA gene at a precise location on its chromosome, and the mecA encodes an alternative penicillin-binding protein with a low affinity for semisynthetic drugs, such as methicillin, nafcillin, and oxacillin (7). Antibiotic-resistant bacteria are an increasing problem worldwide, contributing to longer hospital stays, higher medical costs, and higher rates of morbidity and mortality among patients (24). MRSA strains spread quickly in vulnerable hospitalized (exposed) and healthy non-exposed individuals, profoundly changing the current therapeutic options for the prevention and treatment of *staphylococcal* infections (22, 25).

Carrier health students act as a reservoir for the spread of *S. aureus* to uncolonized susceptible patients. Besides, the chances of the transmission of MRSA from the medical student carriers to the community are a great hazard. University students living in the university dormitory are among the groups living in crowded conditions, which could make them more vulnerable to inadequate care and possibly at higher risk for MRSA colonization. However, little is known about MRSA carriage among pre-clinical undergraduate students living on the university campus or dormitory before their hospital attachment. Therefore, this study aimed to assess the MRSA nasal carriage rate, associated factors, and antimicrobial susceptibility patterns among students at Haramaya University’s College of Health and Medical Sciences (CHMS).

## Materials and methods

### Study area and period

The study was conducted at the CHMS, Haramaya University, Eastern Ethiopia, from 1 July to 30 August 2022. The CHMS is one of the colleges that was established in September 1996 with the objective of training health professionals who contribute to filling the gap in the health professional needs of the country, especially the rural population.

### Study design and population

We carried out an institutional-based cross-sectional study. All undergraduate pre-clinical students from the selected department in the preceding 6 months at the CHMS were recruited. Pre-clinical students who had taken antibiotics within the 10 days before the data collection (26) and participants who had nasal bleeding during the time of collection were excluded. The source population for this study comprised all undergraduate students enrolled during the study period at Haramaya University’s CHMS. On the other hand, all of the selected students from each department were considered the study population during the study period.

### Sample size determination and sampling technique

**Figure 1:**
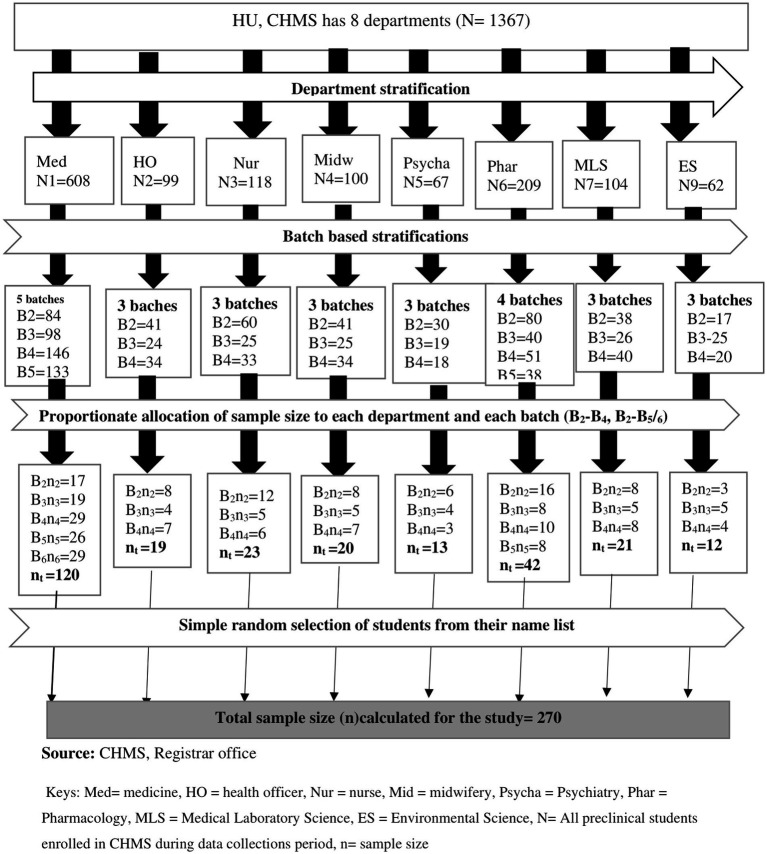
Flow diagram illustrating the sampling procedure of pre-clinical students in CHMS Haramaya University, Harar, Eastern Ethiopia, 2022.

The sample size was determined using a single population proportion statistical formula by considering 95% confidence, p = proportion of *Staphylococcus aureus* in Arba Minch University, Ethiopia (27.1%) (7), and d = the level of precision (0.05). Ten percent of the sample size was added to reduce errors resulting from the likelihood of non-compliance, giving a final sample size of 270. Health and Medical Sciences students were stratified based on academic years; the final sample size was allocated proportionally to each academic year during the study period. A simple random sampling technique was used to select each pre-clinical student who fulfills the inclusion criteria from each departmental batch by using a student roster as a sampling frame that was collected from each department until it reached the allocated sample size (Figure 1).

### Data and sample collections

Data on socio-demographic, behavioral features, clinical information, and associated factors were collected using a structured, pretested questionnaire that was developed after a review of a variety of literature sources (2, 15, 17, 27) through oral interviews of study participants, and nasal swabs were taken from the anterior nares using a sterile cotton-tipped applicator stick saturated with sterile normal saline. The specimens were transported for microbiological analysis to the microbiology laboratory at Haramaya University CHMS within 30 min of collection (28).

### Isolation and identification of *S. aureus*

The nasal swab was inoculated on mannitol salt agar (MSA) and blood agar (Oxoid, United Kingdom) and incubated at 37°C for 24 h. Presumptive *S. aureus* was detected by the visual inspection of golden-yellow colonies on MSA and beta-hemolysis on sheep blood agar. Gram stain, catalase, and tube coagulase tests were used to further identify the isolates (27).

### Antimicrobial susceptibility testing

Antimicrobial tests of *S. aureus* isolates were performed on Muller–Hinton Agar (Oxoid, United Kingdom) using the modified Kirby–Bauer disk diffusion method based on recommendations from the Clinical and Laboratory Standards Institute (CLSI) (28). The suspension of bacterial inoculum, equivalent to 0.5 McFarland standards, was uniformly spread on Mueller–Hinton agar (Oxoid, United Kingdom) plates using a sterile applicator cotton swab. The following antimicrobial disks (Oxoid, United Kingdom) were used: ciprofloxacin (5 mg), clindamycin (2 mg), gentamicin (10 mg), erythromycin (15 mg), chloramphenicol (30 mg), ampicillin (10 mg), tetracycline (30 mg), trimethoprim-sulfamethoxazole (25 mg), and cefoxitin (30 mg). These antibacterial disks were selected depending on local availability, pathogens, and 2020 CLSI recommendations (28). The antibiotic disks were applied to the surfaces of the inoculated Mueller–Hinton agar and incubated at 37°C for 24 h. After incubation, the diameter of the zone of inhibition produced by each antibiotic disk was determined based on the CLSI protocol, and the results were interpreted as sensitive (S), intermediate (I), or resistant (28).

Identification of MRSA: Cefoxitin disk (HiMedia Ltd., Mumbai) was used to identify Methicillin-resistant *Staphylococcus aureus*. The inoculated plate was covered with a cefoxitin disk (30 μg), which was then incubated for 16–18 h at 37°C. An inhibition diameter <22 is indicative of methicillin expression (27).

### Data quality control

Standard operating procedures (SOPs) were strictly followed for each activity in the laboratory. To ensure the validity of the questionnaire, 5% of it was pre-tested at Dire Dawa University. Data collectors were trained regarding all stages of the data collection process. The completeness of each questionnaire is checked by the principal investigator and the supervisors daily. The sterility of uninoculated culture media was checked by incubating 5% of the batch at 37°C overnight, and following incubation, it must remain clear. In addition, standard positive control strains of *Staphylococcus aureus* (ATCC 25923) and MRSA (ATCC 29213) were used for viability and to check the quality of the reagents, culture media, and antimicrobial disks (28). The bacterial suspension’s inoculum density was calibrated using the 0.5 McFarland standard.

### Methods of data analysis

Data were entered into Epi-Data version 4.4.2.1., cleaned, and then exported to the Statistical Package for Social Sciences software (SPSS) version 25 for analysis. The findings were summarized using descriptive statistical tools such as mean, standard deviation, and percentage. Pearson’s chi-square test (*X*^2^) was used to forecast the associations between variables. Variables with a *p*-value of less than 0.05 were considered statistically significant.

### Ethical consideration

Ethical clearance was obtained from the Institutional Health Research Ethics Review Committee of the College of Health and Medical Science, Haramaya University, Haramaya University. Written, and signed consent was obtained from the respondents during the time of the interview. Anonymity and confidentiality were ensured for information obtained from study participants. Finally, the results were reported to the participants for appropriate measurement, particularly MRSA.

## Results

### Socio-demographic characteristics

Of a total of 270 samples analyzed, 65.2% (176/270) were males, with a mean age of 21.32 ± 1.14 and a range of 18–27 years. The majority of the study participants were within the age group of 18–22 years; 85.9, 44.4% (120/270) were medicine students, 28.9% (78/270) were second-year students, and 48.1% lived in groups of six (Table 1).

**Table 1:** Socio-demographic characteristics of pre-clinical students in CHMS, Haramaya University, Harar, Eastern Ethiopia, 2022.

Variable	Category	Frequency (No)	Percentage (%)
Gender	Female	94	34.8
	Male	176	65.2
Age	18–22	232	85.9
	23–27	38	14.1
Department of students	Environmental Health	12	4.4
	Medicine	120	44.4
	Nurse	23	8.5
	Midwifery	20	7.4
	Psychiatry	13	4.8
	MLS	21	7.8
	Pharmacy	42	15.6
	Public Health	19	7.1
Year of Study	Two	78	28.9
	Three	55	20.4
	Four	74	27.4
	Five	34	12.6
	Six	29	10.7
Number of students in the dormitory	Six	130	48.1
	Five	54	20
	Fore	35	13
	Three	40	14.8
	Two	11	4.1

Keys: MLS, Medical Laboratory Science.

### Behavioral characteristics of the study subjects

Only 3.7% (10/270) of the students smoked cigarettes, and all of them were men, 21.1% (57/270) used khat, 38.5% (104/270) drank alcohol, and 25 females were part of this proportion. Approximately 55.6% (150/270) of students washed their hands with soap 0–3 times a day, 41.1% (111/270) had a history of sharing personal items, whereas 64.9% (72/111) shared soap with others, 23% (73/270) picked their noses, 55.6% (150/270) wiped their noses with handkerchiefs, and 38.5% (104/270) cleaned their dorm once a week (Table 2).

**Table 2:** Behavioral characteristics of study participants among pre-clinical students in CHMS, Haramaya University, Harar, Eastern Ethiopia, 2022.

Variable	Category	Frequency *N* (%)
Cigarette smoking	Yes	10 (3.7)
	No	260 (96.3)
Regular contact with the smoker	Yes	28 (10.4)
	No	242 (89.6)
Khat consumption	Yes	104 (38.5)
	No	166 (61.5)
Alcohol use	Yes	57 (21.1)
	No	213 (78.9)
Frequency of hand washing	0–3 times per day	150 (55.6)
	4–7	86 (31.9)
	>7	34 (12.6)
Share personal items	Yes	111 (41.1)
	No	159 (58.9)
Type of materials shared with others	Soap and Towel	25 (9.3)
	Cloth	14 (5.2)
	Soap	72 (26.7)
Habit of nose-picking	Yes	73 (27.0)
	No	197 (73.3)
Way of Cleaning nostrils	Hand kerchief	150 (55.6)
	Fingers	120 (44.4)
Frequency of dormitory cleaning	≥Three times a week	101 (37.4)
	Twice a week	61 (22.6)
	Once a week	104 (38.5)
	No	4 (1.5)

### Clinical-related characteristics

Of the 270 study participants, 4.4% (12/270) had been admitted to the hospital during the previous 3 months, and 94.8% (256/270) had no soft tissue infections. The majority of the students had no history of indwelling medical devices; 92.6, 85.2% (230/270) had no comorbidities, 24.1% (65/270) had used antibiotics in the past 3 months, and 24.24% (19/65) had used ciprofloxacin-type antibiotics (Table 3).

**Table 3:** Clinical characteristics of study participants among pre-clinical students in CHMS, Haramaya University, Harar, Eastern Ethiopia, 2022.

Variable	Category	Frequency *N* (%)
Hospital Admission	Yes	12 (4.4)
	No	258 (95.6)
Soft tissue infection	Yes	14 (5.2)
	No	25 (94.8)
Comorbidity	Gastrointestinal disease	26 (9.6)
	Cardiovascular disease	1(0.4)
	Others (Asthma, RTI, UTI, and DM)	13 (4.8)
	No	230 (85.2)
Indwelling medical device	Yes	20 (7.4)
	No	250 (92.6)
Antibiotics use in the last 3 months	Yes	65 (24.1)
	No	205 (75.9)
Types of antibiotic use	Ciprofloxacin	19 (7.0)
	Amoxicillin	12 (4.4)
	Metronidazole	7 (2.6)
	Doxycycline	7 (2.6)
	Diclo	7 (2.6)
	Unknown	13 (4.8)

Keys: RTIs, Respiratory tract infections; UTIs, urinary tract infections; DM, diabetic mellitus.

### Prevalence of *Staphylococcus aureus* and MRSA

Among a total of 270 study participants, the overall prevalence of *S. aureus* was 35/270 (12.96%) (95% CI: 8.85–16.96); of these, 16/35 (45.7%) were MRSA strains. Therefore, 16/270 (5.9%) (95% CI, 3.09–8.7) of all students were identified as MRSA carriers. Of the total of 35 isolates, 22/270 (8.2%) were isolated from females, of which 5/270 (1.9%) were MRSA. A total of 13/270 (4.8%) isolates were from males, of which 11/270 (4.1%) were MRSA (Table 4).

**Table 4:** Prevalence of *S. aureus* and MRSA among pre-clinical students in CHMS, Haramaya University, Harar, Eastern Ethiopia, 2022.

Variables	Categories	*S. aureus*		MRSA	
		Positive (*n* = 35) *N* (%)	Negative (*n* = 235) *N* (%)	Positive (*n* = 16) *N* (%)	Negative (*n* = 19) *N* (%)
Gender	Male	22 (62.9)	154 (65.5)	11 (68.8)	11 (57.9)
	Female	13 (37.1)	81 (34.5)	5 (31.2)	8 (42.1%)
Age	18–22	28 (80)	204 (86.8)	13 (81.3)	15 (78.9)
	23–27	7 (20%)	31 (13.2)	3 (18.7)	4 (21.1)

### Factors associated with MRSA colonization

Following chi-square analysis, study participants who shared personal items such as soap, cloth, and towels (*X*^2^ = 5.367, *p* = 0.021), smoked cigarettes (*X*^2^ = 36.185, *p* = 0.000), consumed khat (*X*^2^ = 4.130, *p* = 0.042), picked their noses (*X*^2^ = 8.596, *p* = 0.003), and had a history of hospital admission before 3 months (*X*^2^ = 83.11, *p* = 0.00) were significantly associated with MRSA nasal colonization (Table 5).

**Table 5:** Chi-square analysis of MRSA among pre-clinical students in CHMS, Haramaya University, Harar, Eastern Ethiopia, 2022.

Variable	Categories	MRSA		*X*^2^ (*p*-value)
		Positive *n* (%)	Negative *n* (%)	
Gender	Female	11 (6.2%)	165 (93.8)	0.095 (0.758)
	Male	5 (5.3)	89 (94.7)	
Age	18–22	13 (5.6)	219 (94.4%)	0.3071 (0.579)
	23–27	3 (7.9)	35 (92.1)	
Department of students	Environmental health	1 (8.3)	11 (91.7)	1.399 (0.986)
	Medicine	6 (5)	114 (95)	
	MLS	1 (4.8)	20 (95.2)	
	Pharmacy	3 (7.1)	39 (92.9)	
	Public health	2 (10.5)	17 (89.5)	
	Nurse	1 (4.3)	22 (95.7)	
	Midwifery	1 (4.3)	19 (95)	
	Psychiatry	1 (7.7)	12 (92.3)	
Year of study for students	Two	10 (6.7)	139 (93.3)	1.016 (0.602)
	Three	5 (6.2)	76 (93.8)	
	Four	1 (2.5)	39 (97.5)	
Number of students in a dormitory	Six	6 (4.6)	124 (95.4)	1.739 (0.784)
	Five	5 (9.3)	49 (90.7)	
	Four	2 (5.7)	33 (94.3)	
	Three	2 (5)	38 (95)	
	Two	1 (9.1)	10 (90.9)	
The habit of cigarette smoking	Yes	5 (50)	5 (50)	36.185 (0.000)
	No	11 (4.2)	249 (95.8)	
Regular contact with smokers	Yes	2 (7.1)	26 (92.9)	0.083 (0.773)
	No	14 (5.8)	228 (94.2)	
Khat consumptions	Yes	10 (9.6)	94 (90.4)	4.130 (0.042)
	No	6 (3.6)	160 (96.4)	
Alcohols use	Yes	5 (8.8)	52 (91.2)	1.050 (0.306)
	No	11 (5.2)	202 (94.8)	
Sharing personal items	Yes	11 (9)	100 (91)	5.367 (0.021)
	No	5 (3.1)	154 (96.9)	
Types of material shared with others	Soap and Towel	1 (4)	24 (96)	1.342 (0.511)
	Cloth	2()	8 (100)	
	Soap	6 (8.3)	66 (91.7)	
Hospital admissions before 3 months	Yes	8 (66.7)	4 (33.3)	83.11 (0.000)
	No	8 (3.1)	250 (96.9)	
The habit of nose-picking	Yes	6 (16,7)	30 (83.3)	8.596 (0.003)
	No	10 (4.3)	224 (95.7)	
Ways of clean nostrils	Handkerchief	6 (4)	144 (96)	2.246 (0.134)
	Fingers	10 (8.3)	110 (91.7)		Variable	Categories	MRSA	*X*^2^ (*p*-value)	Positive *n* (%)	Negative *n* (%)
Antibiotics used in last 3 months	Yes	3 (4.6)	62 (95.4)	0.264 (0.608)
	No	13 (6.3)	192 (93.7)	
Indwelling medical device	Yes	2 (10)	18 (90)	0.643 (0.423)
	No	14 (5.6)	236 (94.4)	
Frequency of dormitory cleaning	>=three times a week	8 (7.9)	93 (92.7)	1.158 (0.561)
	Twice a week	3 (4.9)	58 (95.1)	
	Once a week	5 (4.6)	103 (95.4)	

Keys: MLS, Medical Laboratory Science.

### Antimicrobial susceptibility patterns

The antimicrobial susceptibility test was performed on 35 *S. aureus* isolates. Out of 35 isolates of *S. aureus*, 19/35 (54.3%) were identified as MSSA, while the rest (16/35) (45.7%) were identified as MRSA. Approximately 91.4% of *S. aureus* were susceptible to clindamycin, 88.6% to chloramphenicol, and 85.7% to both ciprofloxacin and gentamycin. However, 94.3% of *S. aureus* were found to be resistant to ampicillin, 77.1% to erythromycin, and 65.7% to trimethoprim-sulfamethoxazole. Of the 16 MRSA isolates, all of them were sensitive to clindamycin, chloramphenicol, and gentamycin. However, ampicillin and cefoxitin resistance were present in all the MRSA isolates (Table 6).

**Table 6:** Antimicrobial susceptibility pattern of *S. aureus* isolated from nasal swabs of pre-clinical students at CHMS, Haramaya University, Harar, Eastern Ethiopia, 2022.

Antimicrobials		*S. aureus* isolates (*n* = 35) *N* (%)	MRSA (*n* = 16) *N* (%)	MSSA (*n* = 19) *N* (%)
Ampicillin	Susceptible	2 (5.7)	0 (0.0)	2 (10.5)
	Resistant	33 (94.3)	16 (100)	17 (89.5)
Cefoxitin	Susceptible	20 (57.1)	0 (0.0)	11 (57.9)
	Resistant	15 (42.9)	16 (100)	8 (42.1)
trimethoprim-sulfamethoxazole	Susceptible	12 (34.3)	8 (50)	4 (21.1)
	Resistant	24 (65.7)	8 (50)	15 (78.9)
Clindamycin	Susceptible	32 (91.4)	16 (100)	16 (84.2)
	Resistant	3 (8.6)	0 (0.0)	3 (15.8)
Erythromycin	Susceptible	8 (22.9)	8 (50)	2 (10.5)
	Resistant	27 (77.1)	8 (50)	17 (89.5)
Chloramphenicol	Susceptible	31 (88.6)	16 (100)	18 (94.7)
	Resistant	4 (11.4)	0 (0.0)	1 (5.3)
Gentamycin	Susceptible	30 (85.7)	16 (100)	17 (89.5)
	Resistant	5 (14.3)	0 (0.0)	2 (10.5)
Ciprofloxacin	Susceptible	30 (85.7)	11 (68.7)	17 (89.5)
	Resistant	5 (14.3)	5 (31.3)	2 (10.5)
Tetracycline	Susceptible	25 (71.4)	8 (50)	13 (68.4)
	Resistant	10 (28.6)	8 (50)	6 (31.6)

Keys: MSSA, Methicillin susceptible S. aureus; MRSA, methicillin-resistant S. aureus.

### Multidrug resistance of *Staphylococcus aureus* isolates

Of all the isolates, 21 (60%) were multidrug-resistant. Fourteen isolates (66.7%) were resistant to three antimicrobial classes, whereas two (9.5%) were resistant to six antimicrobial classes (Table 7).

**Table 7:** Multidrug resistance nature of *S. aureus* isolates at CHMS, Haramaya University, Harar, Eastern Ethiopia 2022.

Patterns	Antimicrobial agents	Resistance strains	
		Number of *S. aureus*	(%)
For three	AMP, T, CXT	1	66.7
	AMP, CXT, E	2	
	AMP, T, E	6	
	AMP, TS, E	4	
	AMP, CXT, CIP	1	
For four	AMP, T, CL, E	1	9.5
	AMP, T, CXT, E	1	
For five	AMP, GM, CXT, TS, E	3	14.3
For six	AMP, GN, CL, CXT, TS, E	2	9.5
	Total	21	100

Keys: AMP, ampicillin; CIP, ciprofloxacin; GM, gentamycin; TS, trimethoprim-sulfamethoxazole; T, tetracycline; CXT, cefoxitin; E, erythromycin; C, chloramphenicol; CL, clindamycin.

## Discussion

MRSA infections are increasingly recognized worldwide as potentially fatal infections in the population, and they are no longer exclusive to healthcare settings (27). Therefore, using hospital-based infection control strategies alone will not be sufficient to reduce the community’s growing MRSA infection rate. The overall colonization rate of *S. aureus* was 12.96% (95% CI: 8.85–16.96). This is corroborated by a study conducted in Ethiopia (12%) (17), the Democratic Republic of Congo (16.6%) (29), and Nepal (15%) (30). But lower than a report in Ethiopia (22.1%) (22), Tanzania (21%) (21), Nigeria (51.9%) (31), India (29.7%) (6), Taiwan (24.7%) (32), Iran (19.6%) (33), Iraq (17.5%) (25), Thailand (29.7%) (34), Malaysia (31%) (35). In this study, the total prevalence of MRSA was 5.9% (95% CI: 3.09–8.7). This was in agreement with the findings from Jima, Ethiopia (22), Adigrat, Ethiopia (5.8%) (17), Iraq (4.2%) (25), North Korea (3.1%) (36), India (6.1%) (6), and Nepal (4%) (30). However, our prevalence was lower than earlier studies conducted in Nigeria (41.5%) (31) and Iran (13.14%) (33). On the other hand, our result was higher than studies reported from Tanzania (0.3%) (21), China (0.3%) (32), Thailand (0%) (34), and Malaysia (0%) (35). The variation of this prevalence across different studies could be due to differences in personal hygiene and lifestyle traits, sharing of personal items, crowded living environments, direct skin-to-skin contact, hospitalization, study durations, antimicrobial policy, and participant awareness of MRSA (17).

The incidence of MRSA nasal carriage was greater in female students than in male students in our study, but the difference was not statistically significant (*p* = 0.758). This is following an earlier study among clinical samples of *S. aureus* in Ethiopia (17) and other findings conducted by healthy students in Iraq (25) and Afghanistan (2), which shows that gender was not found to be a risk factor for MRSA infection. Additionally, nasal carriage of MRSA was studied in two age groups: 18–22 years and 23–27 years. A total of 7.9 and 5.6% of the students were 23–27 and 18–22 years old, respectively (*p* = 0.579). It is consistent with past findings by Naimi et al. (2) and Legese et al. (17). These indicate that gender and age were not associated factors in the acquisition of MRSA infections.

Numerous studies in different populations showed that socio-demographic traits and other risk factors have a significant role in the nasal carriage of MRSA. Similarly, in the current study, MRSA nasal colonization was significantly associated with a history of cigarette smoking (*p* = 0.000), the habit of consuming khat (*p* = 0.042), nose picking (*p* = 0.003), sharing personal items (*p* = 0.021), and a history of hospitalizations in the previous 3 months (*p* = 0.00). This association may result from frequent hospital visits, which are highly contagious, especially for drug-resistant pathogens; inadequate personal hygiene (the primary mechanism for the auto-transmission of contaminated hands to the nose may be the temporary hand carriage of bacteria on the hands of healthy students); and a history of person-to-person contact with tissue and skin infection (37).

In the antibiotic sensitivity testing, we found that all MRSA isolates were susceptible to clindamycin, chloramphenicol, and gentamycin, which is similar to the findings reported in Adigrat, Ethiopia (17), Nepal (30), Afghanistan (2), and India (6). However, it differs from the result documented in Iraq that indicated all MRSA isolates are sensitive to vancomycin (25). Nonetheless, all MRSA isolates were resistant to ampicillin and cefoxitin, and half of those isolates were also resistant to erythromycin and trimethoprim–sulfamethoxazole, in line with a prior study published in India (6) and Democratic Republic of the Congo, (29). Although the result contradicts previous research in Ethiopia that found a low resistance rate to ampicillin and cefoxitin (48.3%) (17). The cause of this resistance pattern in our findings may be the overuse of these antibiotics for many other infections and the replacement of sensitive strains with resistance strains.

All 35 *S. aureus* isolates were tested for drug susceptibility to 7 commonly used antibiotics. The strains’ resistance to ampicillin, erythromycin, and trimethoprim-sulfamethoxazole is consistent with research done in Ethiopia (17), Jima Ethiopia (22), Tanzania (21), Thailand (34), India (6), and China (32). However, a study conducted in Malaysia revealed no resistance to both cefoxitin and trimethoprim-sulfamethoxazole (35), trimethoprim-sulfamethoxazole in Thailand (34), and low resistance was revealed with trimethoprim-sulfamethoxazole (33%) and gentamycin (2%) conducted in Nigeria (5). Higher gentamycin resistance patterns have been documented in studies carried out in Nigeria (31). This could be brought on by geographical variations in the hospital settings’ approaches to infection prevention and control (2). The most often administered antibiotics in our study area are ampicillin, trimethoprim-sulfamethoxazole, and erythromycin. This might have contributed to the development of resistance to these antibiotics. This study found that *S. aureus* multidrug resistance was quite prevalent. Twenty-one (60%) of the isolates were resistant to three or more antimicrobial classes (38). Thirteen of them (61.9%) were MRSA, and similar susceptibility was observed in Adigrat, Ethiopia (63.6%) (17). This result is significantly higher than the study published in Afghanistan (32%) (2). The possible reasons for this increasing multidrug resistance might be the continuous genetic variation of strains caused by the mutation or cross-transmission of the resistance genetic elements from one bacterium to another, crowded wards, and administration of antibiotics without culture and sensitivity (2, 17).

## Conclusion

This study found a significant rate of MRSA nasal carriage among healthy students when compared to most other similar research. Furthermore, these isolates exhibited high resistance to ampicillin and cefoxitin. Of the MRSA isolates, approximately 61.9% were deemed MDR. It was discovered that there are significant associations between the acquisition of nasal carriage of MRSA and certain student behaviors, including sharing personal items such as clothing, soap, and towels, nose picking, consuming khat, and smoking cigarettes. Hence, routine screening of pre-clinical students is crucial to prevent endogenous infections and the spread of Methicillin-resistant *Staphylococcus aureus*.

### Limitations of the study

The authors did not perform the vancomycin minimum inhibitory concentration due to resource limitations. The species and strain type of MRSA could not be determined using more precise and sensitive molecular techniques. Moreover, phenotypic and genotypic studies are required to determine and elucidate the genetic mechanisms behind antibiotic susceptibilities for the benefit of future researchers. Consequently, further community-based research is needed to find out the prevalence of MRSA in the community.

### Operational definitions

Nasal carriage rate: defined as the prevalence of *S. aureus* and MRSA from the participants isolated from one swab (39).

Susceptible (s): a category denoted by a breakpoint, which implies that isolates with a zone diameter above the susceptible breakpoint are inhibited by generally achievable concentrations of an antimicrobial agent when the recommended dosage is applied to treat the infection site, leading to likely clinical efficacy as determined by laboratory and clinical standards (28).

Resistant (r): a category where a breakpoint indicates that isolates with a zone diameter at or below the resistant breakpoint are not inhibited by the agent at concentrations that are typically achievable using standard dosage schedules (28). In this study, multidrug resistance was defined as resistance to three or more of the tested antimicrobial classes (38).

## Data availability statement

The original contributions presented in the study are included in the article/supplementary material, further inquiries can be directed to the corresponding author.

## Ethics statement

The studies involving humans were approved by Institutional Health Research Ethics Review Committee of the College of Health and Medical Sciences, Haramaya University. The studies were conducted in accordance with the local legislation and institutional requirements. The participants provided their written informed consent to participate in this study.

## Author contributions

FW: Conceptualization, Investigation, Methodology, Project administration, Validation, Writing – original draft, Writing – review & editing, Data curation, Resources, Supervision, Visualization. KU: Conceptualization, Methodology, Supervision, Validation, Visualization, Writing – review & editing. FA: Methodology, Supervision, Validation, Visualization, Writing – review & editing, Data curation, Formal analysis, Funding acquisition, Project administration, Software. KB: Data curation, Formal analysis, Methodology, Project administration, Software, Supervision, Validation, Visualization, Writing – review & editing, Conceptualization. TS: Conceptualization, Formal analysis, Methodology, Project administration, Supervision, Validation, Visualization, Writing – review & editing, Investigation, Writing – original draft. MA: Formal analysis, Investigation, Methodology, Supervision, Visualization, Writing – review & editing. SD: Software, Validation, Writing – original draft, Formal analysis, Investigation, Methodology, Visualization, Conceptualization, Data curation, Funding acquisition. FT: Conceptualization, Data curation, Formal analysis, Investigation, Methodology, Software, Validation, Visualization, Writing – original draft, Project administration, Resources. TT: Conceptualization, Formal analysis, Methodology, Visualization, Writing – review & editing. SM: Conceptualization, Formal analysis, Methodology, Writing – review & editing, Funding acquisition, Investigation, Project administration, Software, Validation, Writing – original draft. HA: Conceptualization, Formal analysis, Methodology, Visualization, Funding acquisition, Project administration, Software, Validation, Writing – original draft, Writing – review & editing.
